# Symmetric and Transitive Registration of Image Sequences

**DOI:** 10.1155/2008/686875

**Published:** 2009-03-24

**Authors:** Oskar Škrinjar, Arnaud Bistoquet, Hemant Tagare

**Affiliations:** ^1^Department of Biomedical Engineering, Georgia Institute of Technology, Atlanta, GA 30332, USA; ^2^School of Electrical and Computer Engineering, Georgia Institute of Technology, Atlanta, GA 30332, USA; ^3^Departments of Electrical Engineering and Diagnostic Radiology, Yale University, New Haven, CT 06511, USA

## Abstract

This paper presents a method for constructing symmetric and transitive algorithms for registration of image sequences from image registration algorithms that do not have these two properties. The method is applicable to both rigid and nonrigid registration and it can be used with linear or periodic image sequences. The symmetry and transitivity properties are satisfied exactly (up to the machine precision), that is, they always hold regardless of the image type, quality, and the registration algorithm as long as the computed transformations are invertable. These two properties are especially important in motion tracking applications since physically incorrect deformations might be obtained if the registration algorithm is not symmetric and transitive. The method was tested on two sequences of cardiac magnetic resonance images using two different nonrigid image registration algorithms. It was demonstrated that the transitivity and symmetry errors of the symmetric and transitive modification of the algorithms could be made arbitrary small when the computed transformations are invertable, whereas the corresponding errors for the nonmodified algorithms were on the order of the pixel size. Furthermore, the symmetric and transitive modification of the algorithms had higher registration accuracy than the nonmodified algorithms for both image sequences.

## 1. INTRODUCTION

The process of aligning images so that the corresponding features can be related is called image registration [1]. Image registration methods have been discussed and classified in books [1–4] and surveys [5–10]. Most registration methods are ad hoc with assumptions often violated in practical applications. This results in a behavior that is often not predictable. A way to reduce the ad hoc nature of registration methods is to require them to satisfy certain properties. Researchers have realized the importance of symmetry and transitivity of registration methods [11–20]. In [11], Ashburner et al. proposed an approximately symmetric image registration method that uses symmetric priors. In [12], Christensen and Johnson proposed a registration algorithm that approximately satisfies the symmetry property. (Christensen and Johnson used the term “inverse consistency” for what we refer to as “symmetry.”) Their idea is to estimate the forward and reverse transformation simultaneously by minimizing an objective function composed of terms that measure the similarity between the two images and the consistency of the forward and reverse transformations. This approach requires maintaining two transformations, computing their inverses and it has a tradeoff among the terms in the objective function. In [13], Rogelj and Kovačič proposed a registration method that uses symmetrically designed forces that deform the two images. The method is approximately symmetric, it requires maintaining forward and backward transformation, but it does not use their inverses. In [14], Škrinjar and Tagare proposed an exactly symmetric registration method that is based on a symmetrically designed objective function, but it requires the computation of the inverse transformation. In [15], Lorenzen et al. proposed an exactly symmetric registration method that is based on a symmetrically designed fluid model. The method uses two transformations whose compositions define the forward and backward transformations in such a way that they are inverses of each other. Beg and Khan in [18] and Avants et al. in [19] used an exactly symmetric registration method that maintains two functions, which when composed appropriately give forward and backward transformations that are exact inverses of each other. In [16], Cachier and Rey analyzed the reasons behind the asymmetry in registration, proposed symmetrized similarity and smoothing energies, but their implementation of the method was not exactly symmetric. In [17], Tagare et al. proposed an exactly symmetric registration method that does not require to maintain both forward and reverse transformations and compute their inverses. Instead, the objective function, which can be based on intensity differences (e.g., mean squared difference, normalized cross-correlation) or distributions (e.g., mutual information, normalized mutual information) is modified such that the method is symmetric and only the forward transformation is needed. Consequently, the objective function has only one term, which avoids the problem of tradeoff among multiple terms. Their implementation is symmetric up to the machine precision. In [20], Christensen and Johnson realized the importance of transitivity of image registration but did not provide a way to satisfy it.

The above methods are either approximately or exactly symmetric but none of them is transitive. In this paper, we propose a method to modify any image registration algorithm such that it is provably symmetric and transitive on an image sequence. Symmetry and transitivity are especially important in motion tracking applications; they insure that a physical point is tracked in the same way regardless of the order in which the images are registered. If there are topological changes present in the image sequence, the two properties can hold only over corresponding regions.

Registration of image sequences has a wide applicability in medical imaging problems. Motion within the body is present at the system level, organ level, tissue level, cellular level, subcellular level, and molecular level. In addition to the normal motion, pathology-induced motion or changes can occur (e.g., osteoporosis, multiple sclerosis, and tumor growth). In both normal and pathology-induced motion or changes, it is often useful to compute the motion, that is, to register image sequences. Such information can improve our understanding of the normal function and diseases as well as help develop better treatments. The presented approach is illustrated on sequences of cardiac MR images, which if accurately registered can provide clinically useful myocardial displacement and strain information. However, the same or similar approach can be used for the registration of any other image sequence.

## 2. METHODS

### 2.1. Notation

Let ℝ denote a set of real numbers and *𝕊* an *N*-dimensional metric space [21]. An *N*-dimensional intensity image is a function *I* : *𝕊* ↦ ℝ. Intensity images will be referred to as just images. Without loss of generality, it is assumed that all the images have the same domain. The set of all images is denoted as *ℐ*. An *N*-dimensional geometric transformation is a function **T** : *𝕊* ↦ *𝕊*. Geometric transformations will be referred to as just transformations. The set of all transformations is denoted as *𝒯*. Let **T**_id_ denote the identity transformation, that is, **T**_id_(**r**) = **r**, ∀ **r** ∈ *𝕊*. Let ∘ denote the composition of transformations.

### 2.2. Image registration operator and its properties

An image registration operator is a function **Γ** : *ℐ* ^2^ ↦ *𝒯*. Ideally, any image registration operator should have the following three properties ∀ *I*, *J*, *K* ∈ *ℐ*.

**Figure 1:**
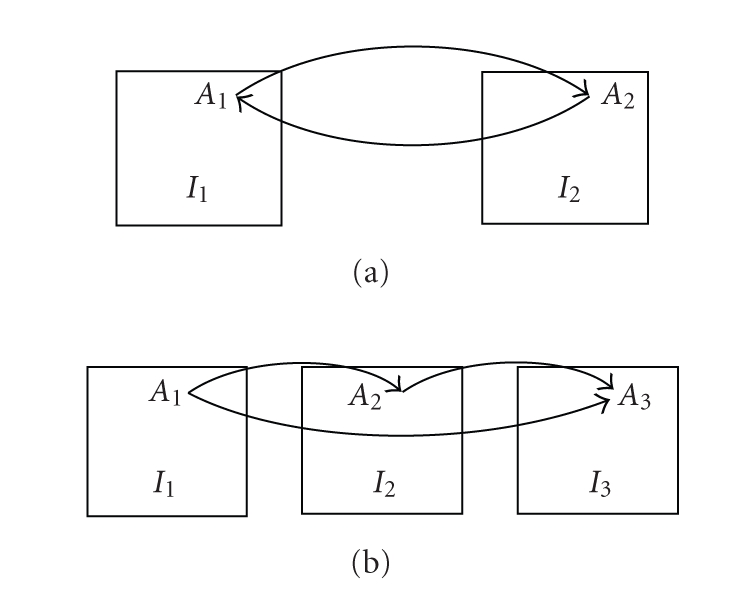
If two points (*A*_1_ and *A*_2_) in two images (*I*_1_ and *I*_2_) correspond, as sketched in (a), then the registration algorithm should associate the two points regardless of the order of images. This is the symmetry property. Let *I*_1_, *I*_2_, and *I*_3_ represent images of the same deforming object taken at three-time points, and let *A*_1_, *A*_2_, and *A*_3_ represent the location of the same physical point in the three images, as sketched in (b). If the registration algorithm, when applied to images *I*_1_ and *I*_2_, associates points *A*_1_ and *A*_2_, and, when applied to images *I*_2_ and *I*_3_, associates points *A*_2_ and *A*_3_, then it should, when applied to images *I*_1_ and *I*_3_, associate points *A*_1_ and *A*_3_. This is the transitivity property.

- *Identity*. An image registration operator, when applied to two identical images should generate the identity transformation. Formally,
  (1) $$\Gamma (I,I)=T_{\text{id}}.$$
- *Symmetry*. The result of the registration should not depend on the order of images, that is, when an image registration operator is applied to two images, the obtained transformation should be the inverse of the transformation obtained when the order of images is reversed. Formally,
  (2) $$\Gamma (J,I)\circ \Gamma (I,J)=T_{\text{id}}.$$
  This is illustrated in Figure 1(a).
- *Transitivity*. For any three images, the generated transformation from the second to the third image composed with the generated transformation from the first to the second image should be equal to the generated transformation from the first to the third image. Formally,
  (3) Γ(J,K)∘Γ(I,J)=Γ(I,K).
  This is illustrated in Figure 1(b).

### 2.3. Reference-based registration

The proposed approach is simple; select a reference image and then perform the registration of any two images from the sequence of images through the reference. The reference can be an image from the sequence of images or an image similar to the images in the sequence. Let the reference be denoted as *R* and let **Γ** represent an image registration operator that does not necessarily have any of the properties from Section 2.2. A new image registration operator **Ψ** is defined as

(4) $$\Psi (I,J)={\left[\Gamma (J,R)\right]}^{-1}\circ \Gamma (I,R),$$

where *I* and *J* are any two images from the sequence. It is assumed that the transformations generated by **Γ** from images in the sequence to the reference image are invertable, that is, that [**Γ**(*J*, *R*)]^−1^ always exists.

**Theorem 1**

**Ψ** *satisfies Identity, Symmetry, and Transitivity*.

**Proof**

Identity holds since

(5) $$\begin{array}{ll} \Psi (I,I) & ={\left[\Gamma (I,R)\right]}^{-1}\circ \Gamma (I,R)\;\text{by}\;\;(4)\\ & =T_{\text{id}}. \end{array}$$

Symmetry holds since

(6) $$\begin{array}{ll} \Psi (J,I)\circ \Psi (I,J) & ={\left[\Gamma (I,R)\right]}^{-1}\circ \Gamma (J,R)\circ {\left[\Gamma (J,R)\right]}^{-1}\\ & \;\circ \Gamma (I,R)\;\text{by}\;\;(4)\\ & ={\left[\Gamma (I,R)\right]}^{-1}\circ \Gamma (I,R)\\ & =T_{\text{id}}\;\text{by}\;\;(4). \end{array}$$

Transitivity holds since

(7) $$\begin{array}{ll} \Psi (J,K)\circ \Psi (I,J) & ={\left[\Gamma (K,R)\right]}^{-1}\circ \Gamma (J,R)\circ {\left[\Gamma (J,R)\right]}^{-1}\\ & \;\circ \Gamma (I,R)\;\text{by}\;\;(4)\\ & ={\left[\Gamma (K,R)\right]}^{-1}\circ \Gamma (I,R)\\ & =\Psi (I,K)\;\text{by}\;\;(4). \end{array}$$

It should be noted that the only requirement for the above result to hold is that [**Γ**(*J*, *R*)]^−1^ exists. This means that the obtained transformations and their inverses do not need to be differentiable. However, a number of registration methods involve regularization terms that use transformation derivatives, in which case the registration operator needs to generate diffeomorphic transformations.

If the Jacobian of the transformation **Γ**(*J*, *R*) is positive then the inverse transformation [**Γ**(*J*, *R*)]^−1^ exists. If the Jacobian of the transformation **Γ**(*J*, *R*) is zero or negative, the inverse transformation does not exist and the reference-based registration operator given by (4) cannot be used or it can be used only over the part of the domain where the Jacobian is positive. Many registration methods control the Jacobian either directly [22–26] or indirectly [12, 27–30] by using a smoothness term that penalizes extreme warps to prevent singularities (zero Jacobian) or folding of the space (negative Jacobian), in which case the inverse transformation exists and the reference-based registration can be used.

## 3. RESULTS

While the result of the previous section holds for any registration operator that generates invertable transformations, here we illustrate the approach on two sequences of cardiac magnetic resonance images using two nonrigid image registration algorithms.

### 3.1. MR protocols

We acquired a 3D anatomical cine MRI scan together with a 3D tagged cine MRI scan of a healthy volunteer and then repeated the acquisitions four months later. The volunteer was a 27-year-old male subject with no history of heart disease. The purpose of the tagged scan was to validate the myocardial deformation recovered from the anatomical scan. The scans were acquired using steady-state free-precession short axis cine imaging (flip angle = 65°, TR = 3.4 ms, TE = 1.7 ms) covering the entire heart on a 1.5 T clinical MRI scanner (Intera, Philips Medical Systems, Best, The Netherlands). All the scans had contiguous short-axis slices with similar field of view and phases covering the entire cardiac cycle and their parameters are given in Table 1. The tags were applied immediately after the detection of the R-wave from the EKG signal and the first frame was acquired at a delay of 15 milliseconds after the R-wave. Two orthogonal sets of parallel planar tags with about 9 mm plane separation were oriented orthogonal to the image planes.

For both scans, for each acquired slice the scanner recorded the rigid body transformation from the scanner coordinate system to the slice. This allowed us to map all the slices to a common coordinate system, that is, to spatially align the anatomical and tagged scans. Similarly, the scanner recorded the start time for each phase (frame) relative to the peak of the R wave, which allowed us to temporally align the anatomical and tagged scans. Since the heart rate, that is, the duration of the cardiac cycle, was not the same for the anatomical and tagged scans, we used relative time (as a percentage of the cardiac cycle) for the temporal alignment.

### 3.2. Myocardial deformation recovery

**Figure 2:**
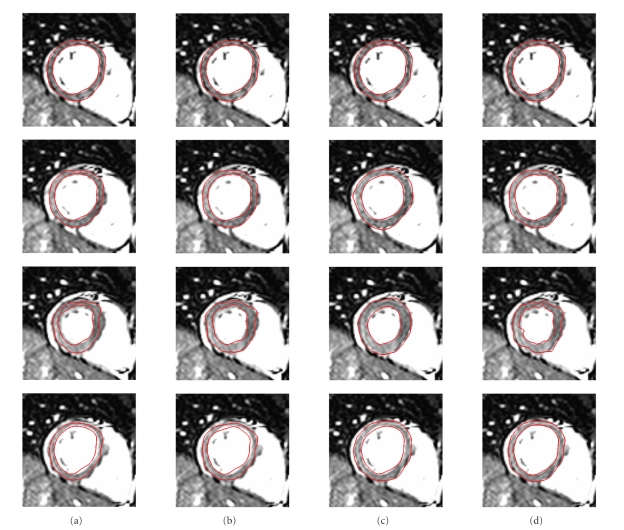
The recovered myocardial deformation for a normal subject over the cardiac cycle (first row: end diastole; third row: end systole) is shown by means of the endocardial and endocardial surface model contours overlaid over a midventricular short-axis MRI slice. The myocardium was segmented in the first frame (shown in the first row), a surface model was generated around the segmented myocardium and the recovered deformation for the rest of the sequence was applied to the surface model. The two red contours represent a cross-section through the surface model. The four columns correspond to (a) sequential recovery by **Γ**_1_, (b) sequential recovery by **Γ**_2_, (c) reference-based recovery by **Γ**_1_, and (d) reference-based recovery by **Γ**_2_. The registration algorithms **Γ**_1_ and **Γ**_2_ and the difference between sequential and reference-based recovery are explained in Section 3.2. Note that the best deformation recovery, that is, the best agreement of the red contours and the edges of the left ventricular wall, was achieved for the reference-based recovery by **Γ**_1_, shown in column (c).

To recover the myocardial deformation, we use thin plate splines (TPS) [31] to represent the transformation between any two frames and then maximize the normalized mutual information [32] to determine the transformation parameters (TPS node positions). We use normalized mutual information since it was shown to outperform several other images similarity measures [33]. Since myocardium is nearly incompressible and its volume does not change by more than 4% over the cardiac cycle [26], we constrain the optimization of the TPS node positions such that the Jacobian of the transformation never deviates from 1 (which corresponds to exact incompressibility) by more than 4%. The details of the method are given in [26]. (The purpose of this section is to illustrate the approach of Section 2.3 (construction of symmetric and transitive registration algorithms from nonsymmetric and nontransitive registration algorithms), and instead of the method given in [26] we could have used any other registration method. For this reason we did not present here all the details of the used registration method and the interested reader is referred to [26].) This registration algorithm we denote as **Γ**_1_, while **Γ**_2_ represents its unconstrained version. Given that near incompressibility is a physical property of the myocardium, **Γ**_1_ is expected to be more accurate than **Γ**_2_. Each of the two operators was used to recover myocardial deformation from the two cardiac MR image sequences in two ways: sequential and reference-based. In the sequential approach, the deformation was recovered from the first to the second frame, then from the second to the third frame, and so on. In the reference-based approach, the deformation was recovered directly from the reference frame to any given frame. Figure 2 shows a short-axis section through the 3D image and 3D LV wall model surface for the sequential and referenced-based recoveries using the two registration operators. 

**Figure 3:**
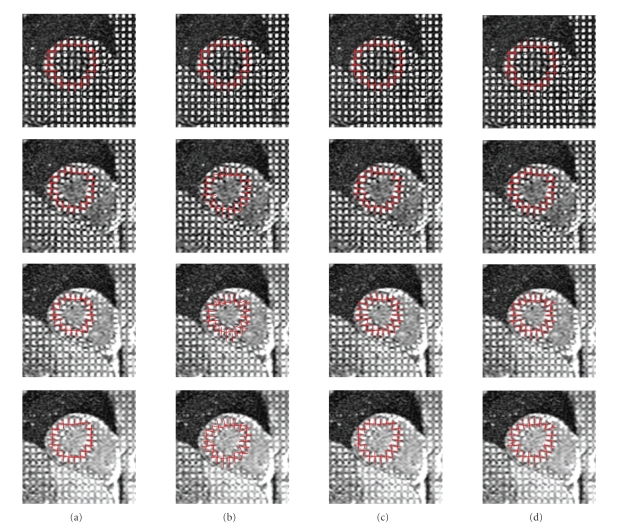
The virtual tag lines and the corresponding short-axis slices of the tagged MRI scan are shown over the cardiac cycle (first row: end diastole; third row: end systole) for (a) sequential recovery by **Γ**_1_, (b) sequential recovery by **Γ**_2_, (c) reference-based recovery by **Γ**_1_, and (d) reference-based recovery by **Γ**_2_. The registration algorithms **Γ**_1_ and **Γ**_2_ and the difference between sequential and reference-based recovery are explained in Section 3.2. The virtual tag lines were manually positioned over the tagged MR image in the first frame (shown in the first row), and then the deformation recovered from the anatomical image sequence was applied to the virtual tag lines and they were overlaid over the tagged MR images in the corresponding frames.

To quantitatively evaluate the deformation recovery accuracy we compared the cardiac deformation recovered from the anatomical cine MRI against the corresponding information from the tagged cine MRI. We developed a tool for interactive positioning of virtual tag planes in tagged MRI scans. The tag planes are modeled as splines that the user can position and deform by moving control points. This allows the user to fit the virtual tag planes to the tagged image as well as to compute tag plane intersections. Once the cardiac deformation is recovered from the anatomical cine MRI using the proposed method, it is applied to the virtual tag planes at ED and then compared to the interactively positioned tag planes in other frames. In each image slice, we compute the distances between the corresponding intersections of orthogonal virtual tag lines (in-slice cross-sections of the virtual tag planes) generated interactively and by the model. This allows for in-plane (short-axis) deformation recovery validation. The out-of-plane (long-axis) deformation is not evaluated with this procedure since the tag planes, being perpendicular to the short-axis image slices, do not contain information about the out-of-plane motion. Virtual tag lines for the sequential and referenced-based recoveries for the two operators are shown in Figure 3. Table 2 contains the distances between corresponding intersections of virtual and real tag lines at end systole, which is the most deformed state relative to end diastole.

### 3.3. Identity, symmetry, and transitivity errors

Let **Ψ**_1_ and **Ψ**_2_ represent the symmetric and transitive modifications of **Γ**_1_ and **Γ**_2_, respectively. Operators **Ψ**_1_ and **Ψ**_2_ are defined by (4) (the end diastole frame is used for *R*), which involves transformation inversion. Since we use TPS for transformation representation and the inverse of a TPS transformation cannot be obtained analytically, we invert the transformation numerically using the Newton-Raphson method for solving nonlinear systems of equations [34]. The numerical error of the computation of the inverse transform is denoted as *ϵ*. If the Jacobian of the transformation is positive then the inverse transformation exists and *ϵ* can be specified to be an arbitrary small positive number, that is, the inverse transformation can be computed with an arbitrary small error. While *ϵ* can be set to an arbitrary small positive value, in practical applications little is gained if *ϵ* is set to a value smaller than two orders of magnitude smaller than the pixel size. The reason for that is that the registration error is on the order of the pixel size, and by setting *ϵ* to one hundredth the pixel size the error of the computation of the inverse transformation is already negligible compared to the registration error, and further reducing *ϵ* does not improve the registration accuracy.

For a given image registration operator **Γ**, we define the following three errors.

- *Identity error* of image *I* is
  (8) $$E_{\text{iden}}=\underset{r\in 𝕊}{\max}∥\Gamma (I,I)(r)-r∥.$$
- *Symmetry error* of images *I* and *J* is
  (9) $$E_{\text{sym}}=\underset{r\in 𝕊}{\max}∥\Gamma (J,I)\left(\Gamma (I,J)(r)\right)-r∥.$$
- *Transitivity error* of images *I*, *J*, and *K* is
  (10) $$E_{\text{tran}}=\underset{r\in 𝕊}{\max}∥\Gamma (J,K)\left(\Gamma (I,J)(r)\right)-\Gamma (I,K)(r)∥.$$

**Figure 4:**
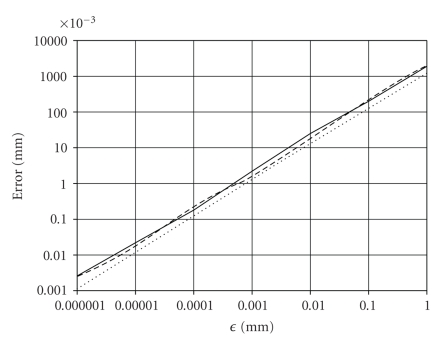
The dependence of *E*_iden_ (dotted), *E*_sym_ (solid), and *E*_tran_ (dashed) on *ϵ* for **Ψ**_1_ is shown in the log-log axes for a representative image, image pair, and image triple, respectively. The three curves represent interpolations of the errors corresponding to *ϵ* of 0.000001 mm, 0.00001 mm, 0.0001 mm, 0.001 mm, 0.01 mm, 0.1 mm, and 1.0 mm. The squared correlation coefficient between *E*_iden_ and *ϵ* is .9998, between *E*_sym_ and *ϵ* is .99997, and between *E*_tran_ and *ϵ* is .9998, indicating a strong dependence of the three errors on *ϵ*.

Identity, symmetry and transitivity errors for **Γ**_1_, **Γ**_2_, **Ψ**_1_, and **Ψ**_2_ are given in Tables 3, 4, and 5, respectively. The errors for **Ψ**_1_ and **Ψ**_2_ in the three tables were computed using *ϵ* = 0.001 mm. The dependence of *E*_iden_, *E*_sym_, and *E*_tran_ on *ϵ* is depicted in Figure 4 for **Ψ**_1_.

## 4. DISCUSSION

Theorem 1 says that reference-based modification of any registration operator satisfies identity, symmetry, and transitivity on an image sequence. While the theorem always holds and the modified registration operator satisfies the three properties exactly, Section 3 demonstrates a practical application of the reference-based registration using a relatively accurate registration algorithm (**Γ**_1_) and its less accurate version (**Γ**_2_). The method was applied to two cardiac cine MRI scans, which are periodic image sequences, but the same conclusions hold in the case of linear image sequences.

As expected, the constrained registration (**Γ**_1_) outperformed its unconstrained version (**Γ**_2_), which can be seen in Figures 2 and 3 and in Table 2 for both sequential and reference-based approaches. The two figures and the table also show that in this case the reference-based registration was more accurate than the sequential registration. The advantage of the sequential registration is that the difference between any two consecutive frames is relatively small, while the reference-based registration deals with larger deformations (e.g., from end diastole to end systole). However, the disadvantage of the sequential registration is that there is accumulation of error from frame to frame, which seems to overweigh the advantage of small frame-to-frame difference. There is no accumulation of error for reference-based registration. The reference-based constrained registration (column (c) in Figures 2 and 3) was more accurate than the other three algorithms (Table 2). The difference in accuracy of the four algorithms is most pronounced at end systole, and it can be seen as the different level of agreement between the model contours and the underlying image in Figure 2 and between the virtual tag lines and the underlying image in Figure 3.

Since **Γ**_1_ and **Γ**_2_ use **T**_id_ as the initial transformation in searching for the transformation that maximizes the normalized mutual information, both operators satisfy (1) exactly and consequently have *E*_iden_ = 0 for any image, as it can be seen in Table 3. Operators **Ψ**_1_ and **Ψ**_2_ involve transformation inversion as defined in (4), which is done numerically with an accuracy of *ϵ*. This is why the identity errors for **Ψ**_1_ and **Ψ**_2_ in Table 3 are approximately equal to *ϵ*.

The symmetry errors (Table 4) for **Γ**_1_ and **Γ**_2_ are approximately 1 mm, while for **Ψ**_1_ and **Ψ**_2_ they are approximately 2*ϵ*. The reason for this is that the evaluation of (9) involves a composition of the operators, each contributing approximately *ϵ* to *E*_sym_ due to the numerical inversion of transformation in (4).

Similarly, the transitivity errors (Table 5) for **Γ**_1_ and **Γ**_2_ are approximately 2 mm, while for **Ψ**_1_ and **Ψ**_2_ they are approximately 2*ϵ*. The reason for this is that the evaluation of (10) involves a composition of the operators, each contributing approximately *ϵ* to *E*_tran_ due to the numerical inversion of transformation in (4).

It should be noted that **Ψ**_1_ and **Ψ**_2_ have similar values for *E*_iden_, *E*_sym_, and *E*_tran_. The reason for this is that the three errors depend only on the accuracy of the computation of the inverse transformation, and not on the registration accuracy (**Ψ**_1_ is more accurate than **Ψ**_2_). In fact, if the inverse transformation could be computed exactly, the three errors would be zero regardless of the registration operator. The three errors scale with *ϵ*, as shown in Figure 4, and they can be made as small as the machine precision. Figure 4 also shows that *E*_iden_ ≈ *ϵ*, *E*_sym_ ≈ 2*ϵ*, and *E*_tran_ ≈ 2*ϵ*. Thus, the reference-based registration slightly worsens the identity error and it significantly improves the symmetry and transitivity errors over the sequential registration.

For the two tested image sequences, the symmetric and transitive registration methods **Ψ**_1_ and **Ψ**_2_ had smaller registration errors than their nonsymmetric and nontransitive counterparts **Γ**_1_ and **Γ**_2_, respectively (Table 2). While all four registration methods had either zero or nearly zero identity errors (Table 3), **Ψ**_1_ and **Ψ**_2_ had very small (nearly zero) symmetry and transitivity errors and **Γ**_1_ and **Γ**_2_ had these errors on the order of the pixel size or even larger (Tables 4 and 5). Thus, in this limited study, very small (nearly zero) symmetry and transitivity errors were accompanied by reduced registration errors. However, to determine if this is always the case one would need to prove it mathematically or at least repeat the experiment on a large number of image sequences.

To simplify the notation, it was assumed that all the images had the same domain, but the same conclusions hold when the domains are different. Furthermore, the method involves the transformation inversion, which is done only once (after both images are registered to the reference), as opposed to the methods proposed in [12, 14–16] that require computing the inverse transformation in each iteration of the optimization.

We used the normalized mutual information as the image similarity measure for the registration algorithms **Γ**_1_ and **Γ**_2_. We repeated all the experiments by using the mutual information, mean square difference, and normalized cross-correlation as alternative image similarity measures and the obtained results were nearly identical to those reported in Section 3 and for this reason they are not included in the paper. These repeated experiments confirm that the conclusions of the paper are not specific to the normalized mutual information.

## 5. CONCLUSION

The reference-based registration of image sequences is provably symmetric and transitive. This conclusion is independent of the images and registration algorithm used. Furthermore, a limited study showed that the reference-based registration was more accurate than the sequential registration, although this cannot be guaranteed to always hold.

## Figures and Tables

**Table 1:** Parameters of the anatomical and tagged MR scans.

Parameter	Scan 1		Scan 2	
	Anatomical	Tagged	Anatomical	Tagged
In-plane resolution [mm]	1.41	1.24	1.44	1.44
Number of slices	12	15	17	17
Slice thickness [mm]	10	10	8	8
Temporal resolution [ms]	38	30	35	30

**Table 2:** The mean (±std) distance [mm] between corresponding intersections of virtual and real tag lines at end systole for the four algorithms for both scans.

	Sequential **Γ**_1_	Sequential **Γ**_2_	Reference-based **Γ**_1_	Reference-based **Γ**_2_
Scan 1	2.3 ± 0.5	2.6 ± 0.6	1.9 ± 0.3	2.2 ± 0.4
Scan 2	2.2 ± 0.4	2.4 ± 0.4	1.7 ± 0.2	2.1 ± 0.3

**Table 3:** Identity errors [mm] for **Γ**_1_, **Γ**_2_, **Ψ**_1_, and **Ψ**_2_ are given for random frames (*F*) of the two cardiac cine MRI scans.

	Scan 1				Scan 2			
*F*	**Γ**_1_	**Γ**_2_	**Ψ**_1_	**Ψ**_2_	**Γ**_1_	**Γ**_2_	**Ψ**_1_	**Ψ**_2_
3	0	0	0.0012	0.0014	0	0	0.0011	0.0014
7	0	0	0.0013	0.0011	0	0	0.0012	0.0011
12	0	0	0.0012	0.0012	0	0	0.0014	0.0013
14	0	0	0.0011	0.0013	0	0	0.0013	0.0011
19	0	0	0.0014	0.0011	0	0	0.0011	0.0012

**Table 4:** Symmetry errors [mm] for **Γ**_1_, **Γ**_2_, **Ψ**_1_, and **Ψ**_2_ are given for random frames (*F*_1_ and *F*_2_) of the two cardiac cine MRI scans.

		Scan 1				Scan 2			
*F*_1_	*F*_2_	**Γ**_1_	**Γ**_2_	**Ψ**_1_	**Ψ**_2_	**Γ**_1_	**Γ**_2_	**Ψ**_1_	**Ψ**_2_
4	12	1.1	1.3	0.0023	0.0018	1.1	0.9	0.0017	0.0021
7	9	0.6	0.8	0.0019	0.0022	0.6	0.7	0.0020	0.0019
14	19	0.8	0.9	0.0021	0.0020	0.8	1.1	0.0015	0.0023
18	6	1.1	1.5	0.0016	0.0015	1.2	1.5	0.0017	0.0016
2	5	0.6	0.5	0.0022	0.0026	0.7	0.8	0.0019	0.0025

**Table 5:** Transitivity errors [mm] for **Γ**_1_, **Γ**_2_, **Ψ**_1_, and **Ψ**_2_ are given for random frames (*F*_1_, *F*_2_, and *F*_3_) of the two cardiac cine MRI scans.

			Scan 1				Scan 2			
*F*_1_	*F*_2_	*F*_3_	**Γ**_1_	**Γ**_2_	**Ψ**_1_	**Ψ**_2_	**Γ**_1_	**Γ**_2_	**Ψ**_1_	**Ψ**_2_
1	3	7	1.6	1.8	0.0026	0.0019	2.2	2.5	0.0022	0.0015
4	9	15	1.7	2.2	0.0022	0.0021	1.8	1.7	0.0018	0.0023
6	12	18	2.2	1.9	0.0016	0.0023	2.5	2.6	0.0023	0.0020
8	2	14	2.2	2.3	0.0017	0.0020	1.9	2.3	0.0017	0.0016
5	6	17	1.9	2.1	0.0019	0.0022	2.5	2.7	0.0021	0.0022
